# PUVA Induced Bullous Pemphigoid in a Patient with Mycosis Fungoides

**DOI:** 10.1155/2017/6134752

**Published:** 2017-04-16

**Authors:** Birgül Özkesici, Saliha Koç, Ayşe Akman-Karakaş, Ertan Yılmaz, İbrahim Cumhur Başsorgun, Soner Uzun

**Affiliations:** ^1^Clinic of Dermatology, Adıyaman University Training and Research Hospital, Adıyaman, Turkey; ^2^Clinic of Dermatology, Kepez State Hospital, Antalya, Turkey; ^3^Department of Dermatology and Venereology, Akdeniz University School of Medicine, Antalya, Turkey; ^4^Department of Pathology, Akdeniz University School of Medicine, Antalya, Turkey

## Abstract

*Background.* Bullous pemphigoid is an autoimmune subepidermal blistering skin disease in which autoantibodies are directed against components of the basement membrane. The disease primarily affects the elderly people and in most of the patients inducing factors cannot be identified. Herein, we report a case of BP that occurred in a patient who was receiving PUVA therapy for the treatment of mycosis fungoides.* Main Observation.* A 26-year-old woman with mycosis fungoides developed blisters while receiving PUVA therapy. On physical examination tense bullae on the normal skin, remnants of blisters, and erosions were observed on her breasts, the chest wall, and the upper abdomen. Histopathological investigations revealed subepidermal blisters with eosinophilic infiltration and in direct immunofluorescence examination linear deposition of IgG along the basement membrane zone was observed. The diagnosis of bullous pemphigoid was also confirmed by ELISA and BIOCHIP mosaic-based indirect immunofluorescence test.* Conclusions.* PUVA therapy is an extremely rare physical factor capable of inducing bullous pemphigoid. So the development of blistering lesions during PUVA therapy may be suggestive sign of a bullous disease such as bullous pemphigoid and it should be excluded with proper clinical and laboratory approaches immediately after withdrawal of PUVA therapy.

## 1. Introduction

Bullous pemphigoid (BP) is an autoimmune subepidermal blistering skin disease, characterized by autoantibodies against structural proteins of the dermal-epidermal junction and clinically presented with cutaneous and/or mucosal blisters or erosions. It usually affects the elderly people. In the most cases, BP occurs sporadically without any obvious precipitation factors. However, the limited BP cases have been reported that are precipitated by UV radiation, medications, vaccination, radiation therapy, thermal burn, amputation stump, incisional hernia scar, and injection [1, 2]. Herein, we report a case of BP that occurred in a patient who was receiving PUVA therapy for the treatment of mycosis fungoides.

## 2. Case Report

**Figure 1:**
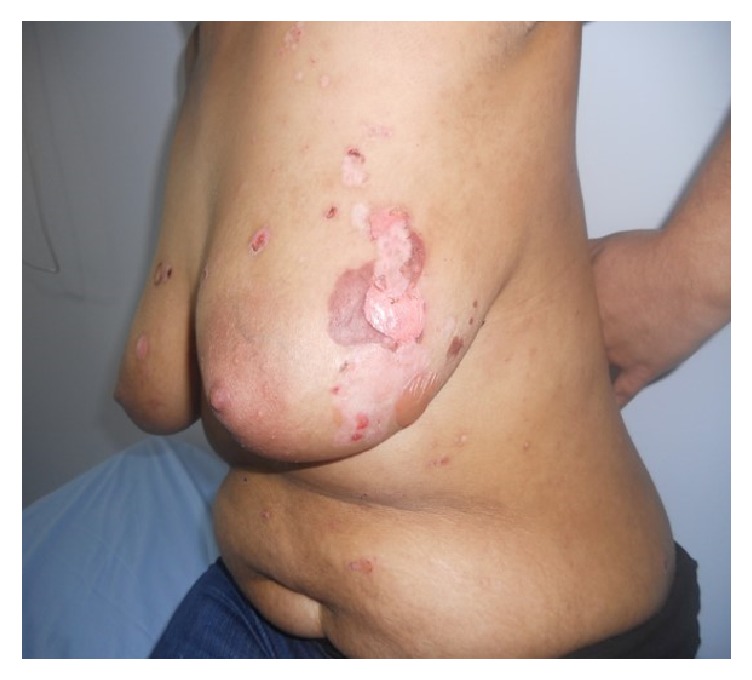
Clinical presentation of the patient; blisters, erosions, and remnants of blisters on her breasts and abdomen.

**Figure 2:**
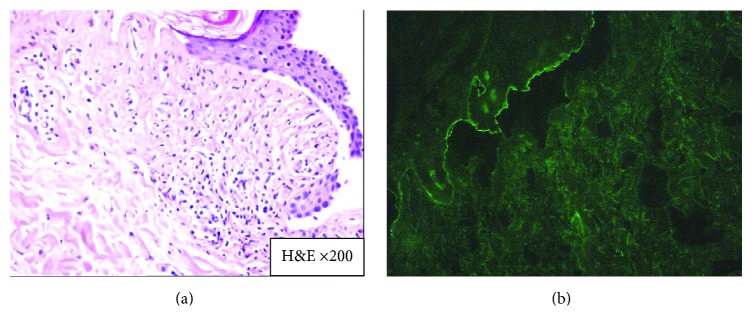
(a) Subepidermal blisters with eosinophilic infiltration (H&E ×200). (b) BIOCHIP demonstrated epidermal deposition on the salt-split skin substrate.

A 26-year-old woman with mycosis fungoides developed blisters while receiving 8-methoxypsoralen and ultraviolet A (PUVA) therapy, at session 17 when the dose of UVA was 7.01 J/cm^2^ (cumulative dose was 70.66 J/cm^2^) (Figure 1). The patient had no history of any other drug use. On physical examination tense bullae on the normal skin, remnants of blisters, and erosions were observed on her breasts, the chest wall, and the upper abdomen. The patch lesions of mycosis fungoides were also present on buttocks and lower abdomen of the patient (stage 1A). PUVA therapy had been stopped and skin biopsies have been taken from a newly formed blister for histopathology and perilesional uninvolved skin for direct immunofluorescence (IF) examination for diagnosis. Histopathological examination showed subepidermal blisters with eosinophilic infiltration (Figure 2(a)) and in direct IF examination linear deposition of IgG along the basement membrane zone was observed. ELISA was positive for anti-BP180 antibody (47 U/mL). Also, BIOCHIP mosaic-based indirect IF test demonstrated a linear deposition of IgG along the basement membrane zone on the primate monkey esophagus, epidermal deposition on the salt-split skin substrate (Figure 2(b)), and anti-BP180 and anti-BP230 positivity on transfected EU90 cells.

**Figure 3:**
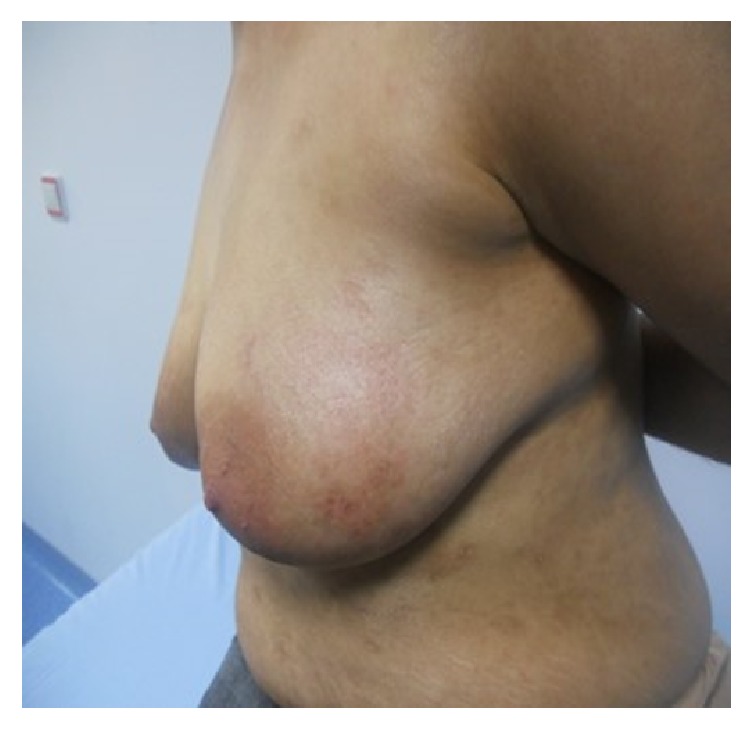
After treatment with 0,05% clobetasol propionate cream. Note the complete healing.

Topical 0,05% clobetasol propionate cream treatment with a dose of 25 gr/day was started after cessation of the PUVA therapy. Disease was gotten under control after 1 week and complete remission was achieved at 3rd week (Figure 3). Then the dose of clobetasol propionate was tapered monthly as 25 mg on alternate days, twice a week, and once a week till it was discontinued after 4 months. Recurrence was not observed during follow-up period for 3 years. After 3 months of the treatment of BP, although patient had narrow band UVB therapy (25 sessions at the cumulative dose of 25.75 joule/cm^2^) for her primary disease, mycosis fungoides, any recurrence of BP was not observed.

## 3. Discussion

The pathogenesis of BP is characterized by tissue-bound and circulating IgG autoantibodies against two components of the hemidesmosome, BP230 and BP180 [1]. The typical clinical presentation is tense blisters frequently concomitant with urticarial plaques. Most cases of BP occur sporadically without any obvious precipitation factors. Local irritation and damage to the skin have all been implicated in the induction of BP. There are limited numbers of reports suggesting that psoralen plus UVA (PUVA) therapy may trigger the development of BP (Table 1) [3–9]. In the majority of these patients PUVA was administered to treat psoriasis. But BP has also been reported previously in a patient with mycosis fungoides [5]. The role of PUVA in the pathogenesis of BP is poorly understood; however, there are some hypotheses that have been proposed. Danno et al. have demonstrated the alterations of keratinocyte surface and basement membrane markers by PUVA therapy [10]. So such changes may induce the production of BP autoantibodies by polyclonal activation of B cells [1]. On the other hand, PUVA may also alter the immunologic reactivity of T-helper and T-suppressor cells that may result in the development of the autoantibodies against native proteins [6]. Inversely, the protective effect of UVB irradiation has been showed by reducing the expression of pemphigoid antigens in organ-cultured normal human skin in a study [11]. In fact, after healing her PUVA induced BP, our patient was treated by UVB for her mycosis fungoides without recurrence of BP.

Although it is difficult to generalize about the prognosis of PUVA induced BP because of the limited numbers of cases [5], it seems that its prognosis is better with mild and transient behavior than idiopathic ones [6].

## 4. Conclusion

Our case indicates that the development of blistering lesions during PUVA therapy may be suggestive sign of a bullous disease such as BP and it should be excluded with proper clinical and laboratory approaches immediately after withdrawal of PUVA therapy.

## Figures and Tables

**Table 1:** Cases of BP due to PUVA therapy.

Cases	Sex	Age (year-old)	Why the treatment of PUVA was given	Type of UVA
1 (2)	Female	45	Vitiligo	Topical PUVASOL
2 (3)	Male	50	Psoriasis	Oral PUVA (methoxsalen)
3 (4)	Male	80	Psoriasis	Oral PUVA (5-MOP)
4 (5)	Female	57	Mycosis fungoides	Oral PUVA (8-MOP)
5 (6)	Female	65	Psoriasis	Oral PUVA (8-MOP)
6 (7)	Female	67	Psoriasis	UVA
7 (8)	Male	58	Psoriasis	PUVA (unspecified)
8 (9)	Male	61	Psoriasis	Oral PUVA (oxsoralen)
9 (present case)	Female	26	Mycosis fungoides	Oral PUVA (8-MOP)
